# Focused ultrasound-mediated blood-brain barrier opening combined with magnetic targeting cytomembrane based biomimetic microbubbles for glioblastoma therapy

**DOI:** 10.1186/s12951-023-02074-z

**Published:** 2023-08-26

**Authors:** Chuanshi He, Zhisheng Wu, Min Zhuang, Xiangyu Li, Shunxu Xue, Songjie Xu, Jinshun Xu, Zhe Wu, Man Lu

**Affiliations:** 1 Department of Ultrasound, Sichuan Clinical Research Center for Cancer, Sichuan Cancer Hospital & Institute, Sichuan Cancer Center, Affiliated Cancer Hospital of University of Electronic Science and Technology of China, Chengdu, China; 2 School of Life Science and Technology, University of Electronic Science and Technology of China, Chengdu, China; 3 Department of Pathology, Sichuan Clinical Research Center for Cancer, Sichuan Cancer Hospital & Institute, Sichuan Cancer Center, Affiliated Cancer Hospital of University of Electronic Science and Technology of China, Chengdu, China

**Keywords:** Microbubble, Focused ultrasound, Glioblastoma, Blood-brain barrier

## Abstract

Glioblastoma is the most common type of brain tumor. Due to the presence of the blood-brain barrier, the effects of chemotherapy have been unsatisfactory. The combination of focused ultrasound and microbubbles to reversibly open the blood-brain barrier is now considered a key factor in improving treatment outcomes of glioblastoma. In this study, we developed bionic drug delivery microbubbles, which in combination with focused ultrasound had an obvious inhibitory effect on glioblastoma. We extracted the brain microvascular cell membranes, combined them with lipid components, and loaded them with superparamagnetic iron oxide and doxorubicin to prepare biomimetic drug delivery microbubbles (FeDOX@cellMBs). We demonstrated that FeDOX@cellMBs retained the intrinsic properties of loading, such as magnetic properties and drug toxicity, both in vitro and in vivo. FeDOX@cellMBs exhibited good tumor targeting and uptake under the combined action of magnetic and focused ultrasound. Importantly, the FeDOX@cellMBs demonstrated excellent internal stability and effectively inhibited tumor growth in orthotopic glioblastoma mice. Finally, organ H&E staining confirmed that FeDOX@cellMBs were safe for use. In conclusion, FeDOX@cellMBs successfully penetrated the blood-brain barrier and effectively inhibited glioblastoma growth under the combined effects of focused ultrasound and magnetic stimulation. These results provide a new approach for the treatment of glioblastoma, with implications for future clinical translation.

**Supplementary Information:**

The online version contains supplementary material available at 10.1186/s12951-023-02074-z.

## Introduction

Glioblastoma is a common type of primary intracranial malignant tumor with a high recurrence rate, high mortality rate, and poor prognosis [1]. The blood-brain barrier (BBB) is composed of tightly packed cerebral microvascular endothelial cells surrounded by peripheral cells, astrocyte termini, and the basement membrane [2]. The BBB protects brain tissue from toxic foreign substances because of the formation of tight junctions between adjacent endothelial cells [3]. However, which the BBB is crucial for brain protection, it also hinders the delivery of chemotherapeutic drugs to brain tissue, thereby limiting the therapeutic effect on glioblastoma [4]. In addition, the brain tumor barrier (BTB), situated between blood vessels and malignant tumors, poses an additional obstacle to the delivery of chemotherapy drugs [5]. Therefore, overcoming the BBB/BTB is a key challenge in improving chemotherapeutic drug delivery for brain tumors.

To address this challenge, considerable efforts have been focused on enhancing drug delivery by overcoming the BBB. Meng et al. [6] conducted clinical trials to demonstrate the clinical translational potential of magnetic resonance (MR)-guided focused ultrasound (FUS) for enhancing trastuzumab delivery in HER2-positive breast cancer. FUS, when combined with intravenously injected microbubbles (MBs), enables repeatable, focal, transient, and non-invasive BBB/BTB opening, enhancing the targeted penetration of therapy [7]. MBs are key catalysts for inducing the BBB opening effect and have gained considerable attention as drug carriers. Encapsulating a destabilizer in MBs offers a key advantage by preventing rapid degradation of the drug, thereby improving therapeutic effectiveness while reducing the required dose [8]. In addition, the release of the encapsulated agent can be controlled during MB destruction triggered by FUS, thus reducing the off-target dose [9]. However, industrially produced lipid MBs have shown low biocompatibility and can be monitored by autoimmune monitoring system during transportation, resulting in excessive and unnecessary losses and even the possibility of immune rejection, which limits their clinical application [10].

Another concern is that despite the success of the targeted opening of the BBB, drug delivery is still passive, relying on the free diffusion of drugs across the barrier. To overcome this, magnetic nanoparticles can be magnetized and made sensitive to external magnetic fields [11]. Thus, magnetic targeting (MT) is used to actively enhance drug deposition at the target site, potentially increasing the therapeutic dose compared to passive diffusion [12]. Therefore, we hypothesized that MT could serve as an active targeting tool to further enhance targeted drug delivery to the open site of the BBB.

**Scheme 1:**
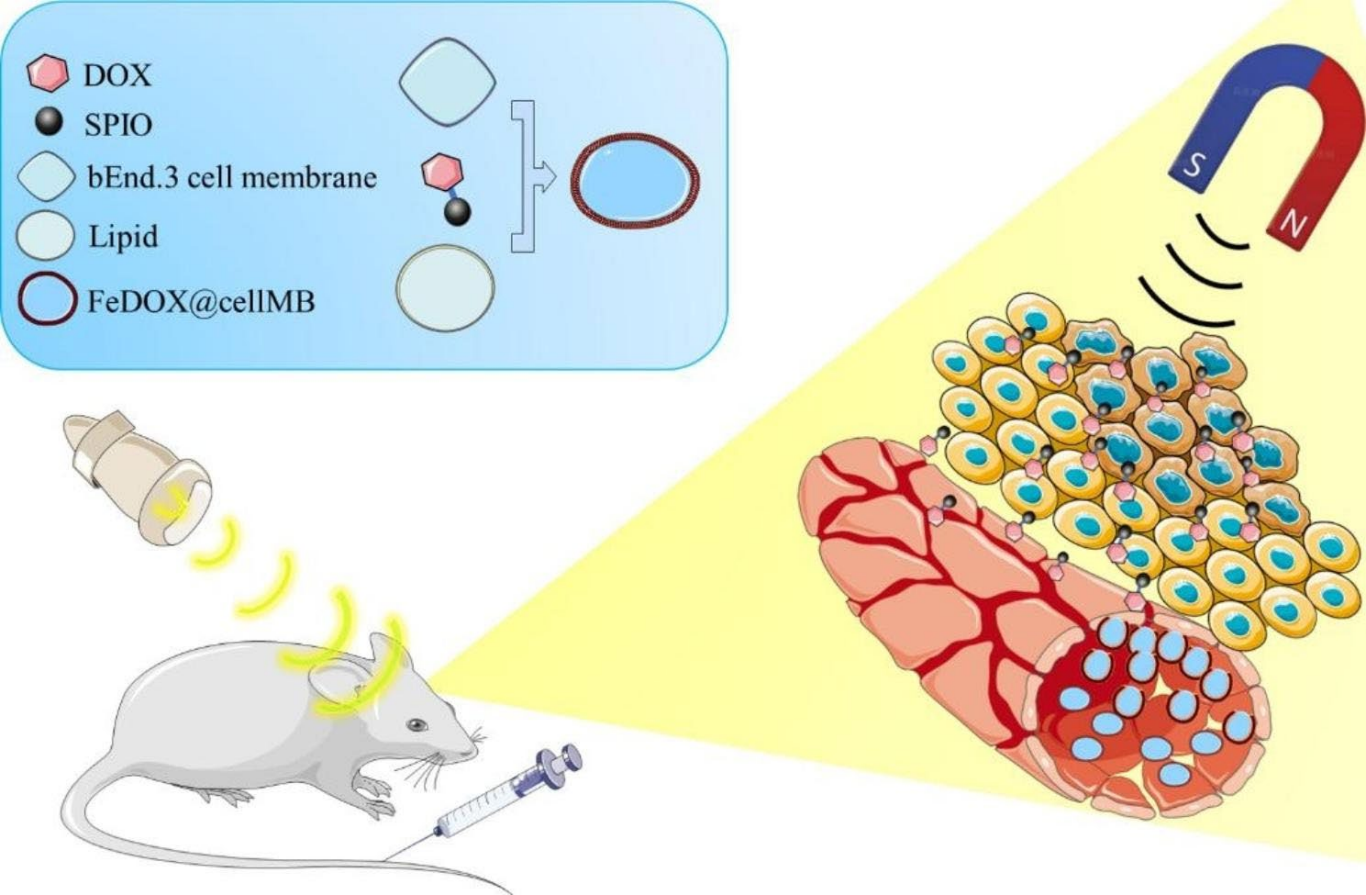
Schematic illustration of FeDOX@cellMBs penetrate BBB and target glioblastoma under the combined effects of focused ultrasound and magnetic

Herein, we present the development of biomimetic MBs produced from the cell membranes of cerebral microvessel cells and lipids. In addition, superparamagnetic iron oxide (SPIO) nanoparticles were directly coupled to doxorubicin (DOX) and embedded in biomimetic MBs (FeDOX@cellMBs). By applying FUS and MT simultaneously, we demonstrated that FeDOX@cellMBs triggered BBB opening and allowed drugs to penetrate brain tumors in an actively targeted manner (Scheme 1). Furthermore, we found that biomimetic MBs, prepared using membrane and lipid fusion techniques, enhance the stability of drugs in vivo and lead to improved therapeutic outcomes.

## Materials and methods

### Cell culture

Glioblastoma cell line GL261 and cerebral microvascular cell line bEnd.3 were purchased from the American Type Culture Collection (ATCC, Manassas, VA, USA). GL261 and bEnd.3 cells were cultured in Dulbecco modified Eagle’s medium (DMEM) containing 10% fetal bovine serum (FBS), 1% penicillin and streptomycin and incubated in a tissue culture incubator at 37℃ and 5% CO_2_.

### Preparation of SPIO-DOX complex (FeDOX)

SPIO nanoparticles (average diameter, 50 nm; concentration, 2 mg/mL) were purchased from Xi’an Delta Biological Technology Co., Ltd. Doxorubicin was purchased from MedChemExpress (Monmouth Junction, NJ, USA). The SPIO solution was replaced with deionized distilled water and mixed with the DOX solution under sonication. SPIO particles and DOX were incubated in a bath at 37 °C for 4 h through the natural reaction, and then phosphate buffered saline (PBS) and magnetic precipitation were slowly added to collect the FeDOX complex. To separate the well-conjugated FeDOX complex and unconjugated DOX molecules, the collected FeDOX solution was centrifuged at 11,000 ×g for 3 min, and then resuspended in PBS.

### Preparation of cell membrane modified microbubbles (cellMBs) and FeDOX@cellMBs

The lipid shells of MBs were synthesized using 1,2-distearoyl-sn-glycero-3-phosphocholine (DSPC), 1,2-distearoyl-snglycero-3-phospho-rac-glycerol sodium salt (DSPG) and 1,2-distearoyl-sn-glycero-3-phosphoethanolamine-N-[methoxy(poly(ethyleneglycol))-2000] (DSPE-PEG2000) purchased from Xi’an Ruixi Biological Technology Co., Ltd with a molar ratio of 21:21:1; the three substances dissolved with chloroform while protected from light, and then the chloroform was removed using an evaporator (Yarong, Shanghai, China). The formed dried lipid film was mixed with glycerol PBS, and then the gas in the solution was exhausted and perfluoropropane (C_3_F_8_) was added. Finally, the mixture was shaken for 60 s using an agitator to obtain MBs.

The method used for synthesizing CellMBs was similar to that used for synthesizing MBs, except that the extracted cell membrane was added after rotational evaporation. Briefly, sufficient cells were cultured and washed twice with PBS after digestion with pancreatic enzymes, and the supernatant was discarded by centrifugation at 1200 ×g. The cells were resuspended in hypotonic buffer, and a protease inhibitor was added. After the cell suspension was homogenized 50 times on ice, the supernatant was retained after centrifugation at 4℃ and 3200 ×g for 5 min. This step was repeated twice. All the supernatants were collected, centrifuged at 4℃ at 20,000 ×g for 20 min, and the precipitate was discarded. The supernatant was collected, centrifuged at 100,000 ×g at 4℃ for 1 h, and then pelleted by PBS. The collected cell membranes were added to glycerol PBS containing dissolved lipid membranes, and the subsequent steps were identical to those followed for the synthesis of MBs. In addition to FeDOX (4 mg), FeDOX@cellMBs were obtained in the same manner. The structure of the cellMBs was observed using confocal laser scanning microscope (CLSM, Leica, Germany).

### Transmission electron microscopy (TEM)

A few prepared MBs were dropped on the double-sided copper mesh with a carbon support film, and after drying naturally, their size and shape were observed under TEM (H-600, Hitachi, Tokyo, Japan), and photographs were taken. For FeDOX@cellMBs, the loading of FeDOX complexes was verified simultaneously.

### Nanoparticle tracking analysis (NTA)

The concentration and size distribution of the MBs and FeDOX@cellMBs were analyzed using Nanoparticle tracer analyzer (Malvern, Malvern, UK) and the corresponding software ZetaView 8.05.14. PBS was used to dilute the MB and FeDOX@cellMB samples to measure the particle size and concentration. NTA measurements were recorded and analyzed. The temperature was maintained at approximately 25 °C and the PH at 7.0.

### Evaluation of FeDOX encapsulation efficiency

FeDOX@cellMBs were resuspended with PBS and treated with a self-developed high-intensity focused ultrasound instrument (1.1 MHz, 1 V, Fig. S1) for 5 min to destroy FeDOX@cellMBs. The supernatants containing free FeDOX were collected by centrifugation. The precipitate was collected and resuspended in PBS to obtain MBs containing FeDOX. Free FeDOX and encapsulated FeDOX complexes were nitrified, and inductively coupled plasma-atomic emission spectrometry (ICP-AES) was estimated. The formula for calculation the encapsulation efficiency is:

Encapsulation efficiency (%) = $$\frac{Wencapsulated FeDOX}{Wencapsulated FeDOX+Wfree FeDOX}$$×100%

W_encpsulated FeDOX_ is the amount of encapsulated FeDOX, and W_free FeDOX_ is the amount of free FeDOX.

### Contrast-enhanced ultrasonography (CEUS) of FeDOX@cellMBs

A mold with 2% (w/v) agarose was prepared for contrast-enhanced ultrasonography (CEUS), and three small holes with a diameter of 1.0 cm were made in the mold as the sample wells. CEUS was performed using a clinical US imaging system (EPIQ7 US System, PHILIPS, Netherlands) with a high-frequency linear probe (frequency: 10 MHz). An aliquot of 500 µL FeDOX@cellMBs was diluted 10 times with PBS into the three sampling wells, the probe position was adjusted so that the three sampling wells could be displayed completely at the same time, and an US image was recorded every 10 min.

### Stability of FeDOX@cellMBs

The clinical US imaging system EPIQ7 US system (PHILIPS, Netherlands) with a high-frequency linear probe (frequency: 10 MHz) was used to verify the acoustic stability. FeDOX@cellMBs were diluted 10 times with PBS, added to the agarose mold, the ultrasonic probe was placed on the model for continuous examination, and images were taken every 10 min. Examination was performed 1 h later, and the contrast enhancement of the FeDOX@cellMBs was quantified using the contrast-to-noise ratio (CNR).

The method for assessing serum stability of FeDOX@cellMBs was as follows: FeDOX@cellMBs were diluted 10 times with 10% FBS, added into 96-well plate and incubated in 37℃ with 5% CO_2_. The FeDOX@cellMBs were collected at 0 min, 30 min, 1 h, 2 h, 6 h, 12 and 24 h in microcentrifuge tubes and centrifuged to separate the sediment from the supernatant. Finally, IVIS Lumina II in vivo optical imaging (Caliper, USA) was used to observe FeDOX leakage over time.

### Cellular uptake of FeDOX@cellMBs by GL261 cells

After successful establishment of the BBB model in vitro, GL261 cells were seeded into the receptor chamber with a glass lid at the bottom. After 24 h of culturing, fresh serum-free medium containing FeDOX@cellMBs was added to the donor cavity with or without the process of MT using a 0.48 T permanent magnet (Chengdu Zhiyue Shuangchen Biotechnology Co., Ltd.) under the cell dish for 10 min, with or without FUS administration (1.1 MHz, 1 V) for 5 min according to the groups. After 6 h of incubation, the recipient cavity was washed thoroughly with PBS. The cells were fixed with a 4% paraformaldehyde solution at room temperature for 20 min. An aliquot of 500 µL of 500 nmol/L DAPI solution was added to stain the nuclei for 15 min. Finally, DOX accumulation in GL261 cells was observed using CLSM.

### Cell counting kit-8 (CCK-8)

A CCK8 assay was used to evaluate cell growth. GL261 cells (4 × 10^3^ cells/well) were seeded into 96-well plates. PBS, DOX, FeDOX, cellMBs, and FeDOX@cellMBs were incubated with the cells for 2 h with or without MT using a 0.48 T permanent magnet under the cell dish for 10 min, and with or without FUS (1.1 MHz, 1 V) for 5 min. After 48 h, 10 µl CCK8 solution was used to each well, incubated for 1 h, and the absorbance was measured at 450 nm.

### Orthotopic GBM animal model

An orthotopic GBM animal model was established using female C57BL/6 mice (6–8 weeks old) purchased from Beijing Vital River Laboratory Animal Technology Co., Ltd. Mice were anesthetized, their heads were immobilized with a stereotactic fixation device, and after skin preparation and disinfection, a median incision was made on the scalp to expose the bregma. A small dental drill was then used to drill a hole (approximately 1.0 mm diameter) 1.0 mm in front of the bregma and 2.0 mm outside the right sagittal suture. A total of 2 × 10^5^/µL GL261 glioma cells were resuspended in 10 µl phosphate buffered saline (PBS) and slowly injected at a depth of 3.0 mm into the above hole with a microinjector. Finally, the incision was closed using medical sutures. The mice were treated 1 week after tumor implantation.

All animal experiments followed the guidelines for animal care and use of experimental animals published by the Institutional Animal Care and Use Committee of Sichuan Cancer Hospital (Grant No. SCCHEC-04-2020-004).

### In vivo magnetic and FUS experimental setup

FUS was transcranially applied to the tumor location (sine wave, PRF: 1100 kHz, acoustic amplitude: 1 V, sonication duration: 4 min). An MT with a 0.48 T permanent magnet was placed tightly at the tumor location for 3 h.

### Biodistribution of FeDOX@cellMBs in orthotopic GBM mice

FeDOX@cellMBs (100 ul, 10 times diluted with 0.9% normal saline) were injected into orthotopic GBM mice through the tail vein and treated with high intensity FUS. The IVIS Lumina II in vivo optical imaging (Caliper) was used to observe the biodistribution at 6 and 12 h in mice. The mouse brain and fluorescence intensity were analyzed semi-quantitatively, and the targeting efficiency of FeDOX@cellMBs was calculated using the following formula:

Target efficiency = (Brain fluorescence intensity/body fluorescence intensity) × 100%.

After injection of FeDOX@cellMBs into the tail vein and treatment with high-intensity FUS for 2, 4, 6, and 12 h, the mice were sacrificed, and the heart, liver, spleen, lung, kidney, and brain were separated. The accumulation of FeDOX@cellMBs in the brain tissue and other organs was observed using the IVIS Lumina II in vivo optical imaging (Caliper).

Finally, the tissues were soaked in a 4% paraformaldehyde solution for 24 h, and paraffin sections were prepared to observe the distribution of FeDOX@cellMBs.

### In vivo anti orthotopic GBM activity of FeDOX@cellMBs

We used a self-developed high-intensity FUS instrument to induce the cavitation effect, open the blood-brain barrier (BBB), and simultaneously release the drugs. After anesthesia, the orthotopic GBM mice were shaved off the fur on top of their heads, placed in a prone position, and fixed on the operating platform. The probe was adjusted to the tumor site of the mice, the corresponding drugs were administered through the tail vein, and FUS was initiated. During the MT operation, a permanent magnet was placed tightly on the scalp of the mice for 3 h.

Orthotopic GBM mice were randomly divided into three groups: 1) FUS sonication and MT following injection of FeDOX@cellMBs (FeDOX@cellMBs + FUS + MT group, n = 3); FUS sonication and MT following injection of the FeDOX complex and cellMBs (FeDOX + cellMBs + FUS + MT group, n = 3); and FUS sonication following injection of PBS (PBS + FUS group, n = 3).

The mice were treated once every other day for 14 days. Tumor changes were observed using the IVIS Lumina II in vivo optical imaging (Caliper) to reflect the treatment effect.

### Statistical analysis

All data are expressed as mean ± standard deviation (SD). Differences between the test groups were compared using two-tailed paired Student’s t-test, one-way analysis of variance (ANOVA), and Tukey’s post-hoc test. All analyses were performed using SPSS software (IBM, Armonk, NY, USA) and GraphPad 6.0. Statistical significance was set at p < 0.05.

## Results

### Characterization of FeDOX@cellMBs

**Fig. 1:**
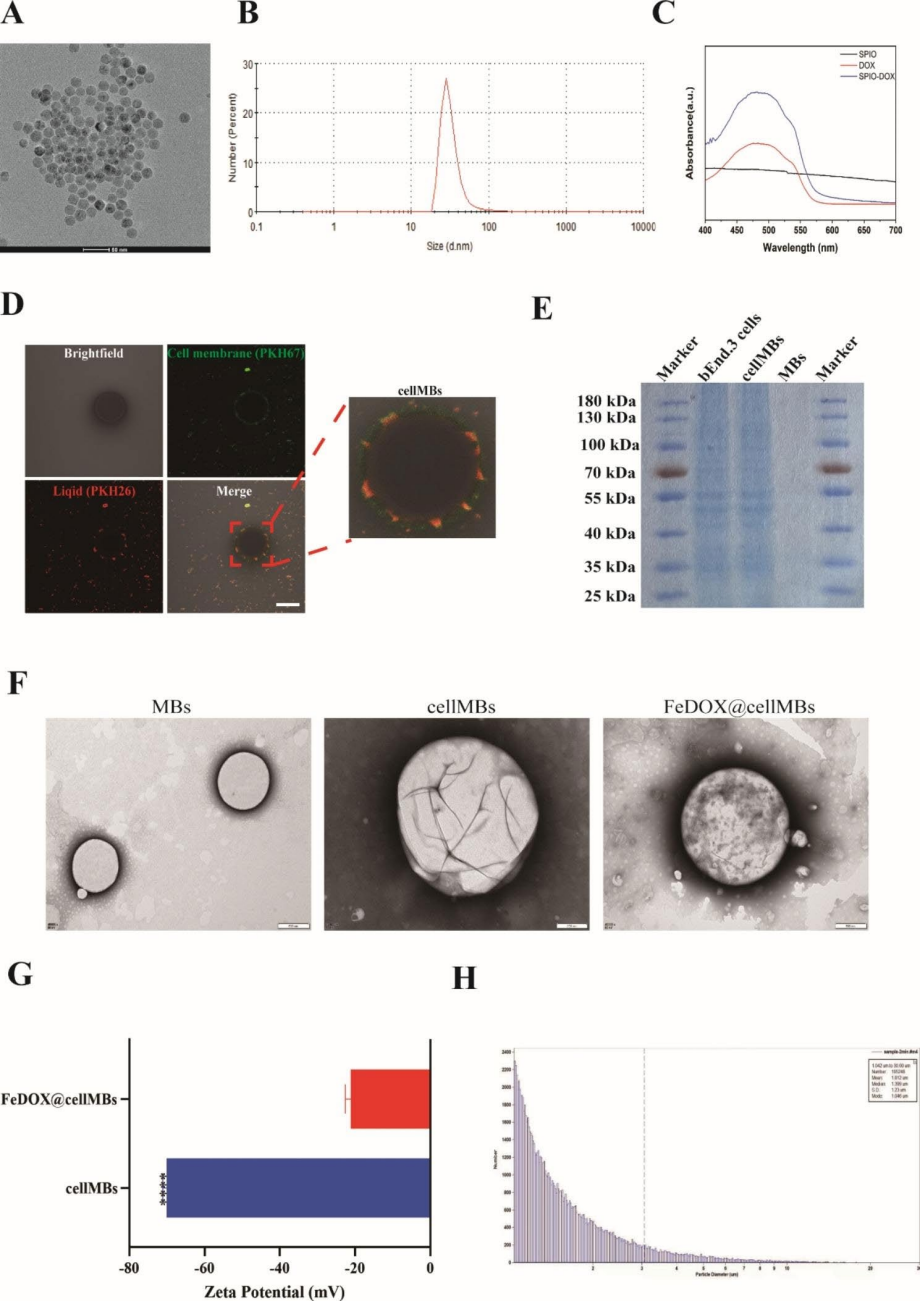
Characteristics of FeDOX@cellMBs. (**A**) TEM images of FeDOX. (**B**) Size distribution of FeDOX. (**C**) UV–vis spectra of FeDOX and its constituents. (**D**) Cell membranes of cerebral vascular endothelial cells were extracted and fused with lipid components to prepare biotype ultrasonic microvesicles (cellMBs). Scale bar = 1 μm. (**E**) SDS-PAGE protein analysis of bEnd.3 cell, cellMBs and MBs. (**F**) TEM images of MBs, cellMBs and FeDOX@cellMBs. (**G**) Zeta potential of FeDOX@cellMBs and cellMBs. (**H**) Size distribution and concentration of FeDOX@cellMBs. (n = 3, x ± SD. **** P < 0.0001)

FeDOX was prepared using SPIO particles and the natural reaction between the amino and carbonyl groups of DOX [13]. The TEM results revealed the morphology and structure of FeDOX; the particle size was generally below 50 nm, which was also confirmed by the particle size detection results (Fig. 1A, B). The DOX load was found to be 11.3 ± 3.7% using UV-VIS spectroscopy (Fig. 1C). These results confirmed the successful synthesis of the FeDOX complex.

The cell membranes of cerebral vascular endothelial cells were extracted and fused with lipid components to prepare biotype ultrasonic microbubbles (cellMBs) to load the FeDOX complex (FeDOX@cellMBs). We first stained the cell membrane and lipid components separately and observed the structure of the biotype microbubbles using confocal microscopy (Fig. 1D). Interestingly, our results confirmed good fusion of the two lipid components. Gel electrophoresis experiments showed that the protein bands of cellMBs were the same as those of the cell membrane of bEnd.3 cells (Fig. 1E), indicating that the surface of cellMBs was successfully coated by the cell membrane of bEnd cells. Next, we loaded the FeDOX complex into cellMBs. Light microscopy and TEM were used to better observe the morphological structure (Fig. 1F and Fig. S2). The loading of CellMBs with the FeDOX complex was verified by measuring the zeta potential (Fig. 1G). The zeta potentials of cellMBs and FeDOX@cellMBs were − 22.15 ± 0.83 mV and − 70.09 ± 0.57 mV, respectively. These results confirmed the successful loading of the FeDOX complex into cellMBs. Subsequently, NTA results confirmed that the particle size of the FeDOX@cellMBs remained at the micron level, meeting the standards for ultrasonic microbubbles (Fig. 1H). In summary, these results confirm that the FeDOX complex and biotype ultrasonic MBs were synthesized and effectively loaded, resulting in the successful preparation of FeDOX@cellMBs.

### In vitro antitumor cytotoxicity of FeDOX@cellMBs

**Fig. 2:**
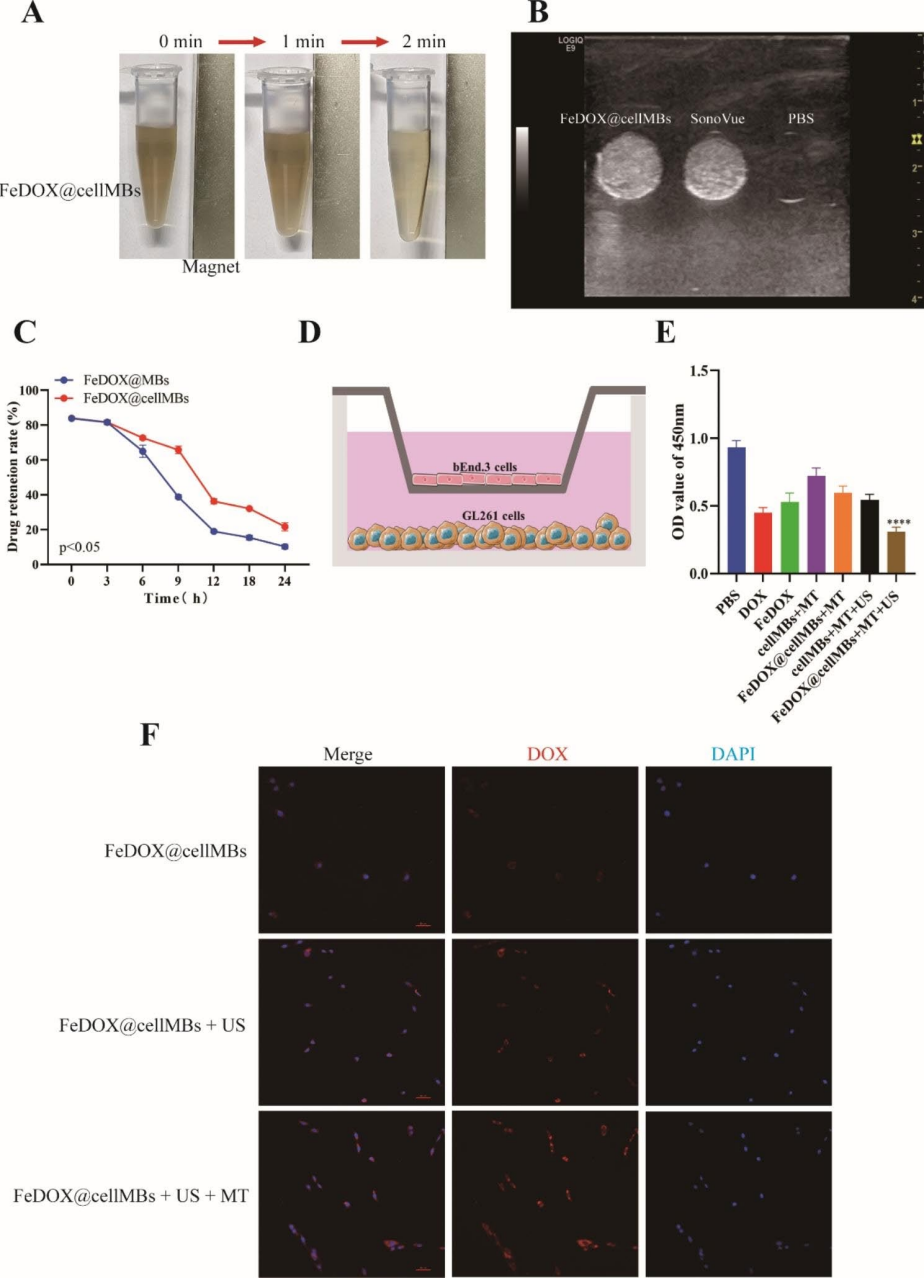
Antitumor cytotoxicity of FeDOX@cellMBs. (**A**) Saturation magnetization of FeDOX@cellMBs. (**B**) Ultrasound imaging of PBS, SonoVue and FeDOX@cellMBs. Scale bar = 1 cm. (**C**) Serum stability of FeDOX@MBs and FeDOX@cellMBs. (**D**) Schematic illustrations of in vitro BBB model. (**E**) Cell viability after treatment. (**F**) The accumucation of FeDOX@cellMBs in GL261 cells. Scale bar = 50 μm. (n = 3, x ± SD. **** P < 0.0001)

To confirm the function of FeDOX@cellMBs, we carried out a series of in vitro functional experiments. Based on the magnetic properties of SPIO, we confirmed the magnetization properties of FeDOX@cellMBs through in vitro magnetic field experiments. When the magnetic field was applied for 2 min, FeDOX@cellMBs were attracted by a permanent magnet, indicating that FeDOX@cellMBs have high magnetization ability (Fig. 2A). In contrast to the clinical ultrasonic contrast agent, FeDOX@cellMBs have good contrast function under ultrasound, indicating that FeDOX transfer and cell membrane addition do not allow imaging of the microbubbles themselves (Fig. 2B). In addition, compared to ordinary lipid microbubbles, FeDOX@cellMBs exhibited better serum stability (Fig. 2C). These results indicated that the synthesis of FeDOX@cellMBs did not affect the basic functions of the individual components and was more stable.

Next, we verified the cellular uptake and cytotoxicity of the FeDOX@cellMBs using an in vitro BBB model (Fig. 2D). Under the combined action of ultrasound and magnetic field, FeDOX@cellMBs permeated better through the BBB and were taken up more efficiently by GL261 cells compared to the control group (Fig. 2F). The CCK8 experiment confirmed that FeDOX@cellMBs had the greatest killing effect on GL261 cells when the ultrasound and magnetic field were applied together (Fig. 2E). In conclusion, the combined effect of ultrasound and magnetic field can maximize the physiological function of FeDOX@cellMBs and achieve targeted therapy for GBM.

### The biodistribution and BBB-opening capability of FeDOX@cellMBs

**Fig. 3:**
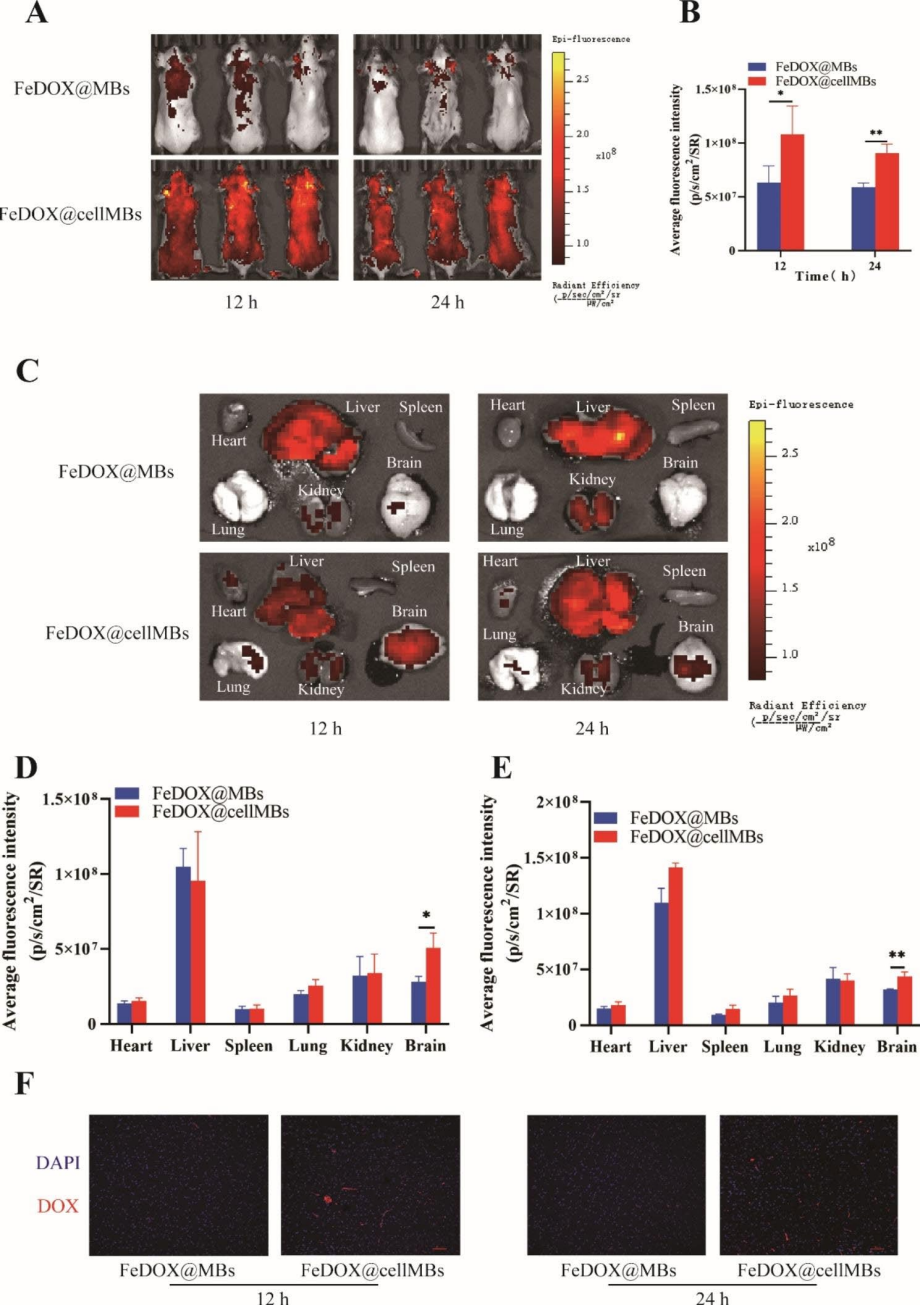
Biodistribution of FeDOX@cellMBs in orthotopic GBM mice. (**A**) Distributions of FeDOX@MBs and FeDOX@cellMBs visualized by IVIS Lumina II in vivo optical imaging. (**B**) The fluorescence intensity of FeDOX@MBs and FeDOX@cellMBs to brain in orthotopic GBM mice. (**C**) Fluorescence in intact organs in orthotopic GBM mice 12 and 24 h after intravenous injection of FeDOX@MBs and FeDOX@cellMBs. (**D**) Semi-quantitative statistic results of fluorescence intensity in main organs collected 12 h after injection of FeDOX@MBs and FeDOX@cellMBs. (**E**) Semi-quantitative statistic results of fluorescence intensity in main organs collected 24 h after injection of FeDOX@MBs and FeDOX@cellMBs. (**F**) Distribution of FeDOX@MBs and FeDOX@cellMBs in GBM tissue of orthotopic GBM mice observed by CLSM. (n = 3, x ± SD. * P < 0.05, ** P < 0.01)

We intravenously injected FeDOX@MBs and FeDOX@cellMBs, and after 12 and 24 h, we observed that FeDOX@cellMBs accumulated in higher amounts in GBM mice in situ than ordinary lipid microbubbles (Fig. 3A). Fluorescence intensity was still observed in the brains of mice 24 h after injection, indicating that FeDOX@cellMBs were more stable in vivo (Fig. 3B). Upon dissecting the organs of mouse after 12 and 24 h (Fig. 3C), we foung that the accumulation of FeDOX@cellMBs in the brain was higher than that of FeDOX@MBs at both time points. This indicates that FeDOX@cellMBs show good brain targeting in vivo under the combined action of ultrasound and magnetic field (Fig. 3D, E). In addition, DOX accumulation in mouse brain tumor tissues was observed at both time points. The results also confirmed that the amount of FeDOX@cellMBs that accumulated in the brain was higher than that of the FeDOX and cellMB mixtures, indicating that FUS achieved BBB opening through the microbubbles (Fig. 3F). In conclusion, under the combined action of FUS and a magnetic field, FeDOX@cellMBs can open the BBB, thus increasing drug concentration in the brain and improved targeting of brain tumors.

### The therapeutic effect of FeDOX@cellMBs on orthotopic GBM mice

**Fig. 4:**
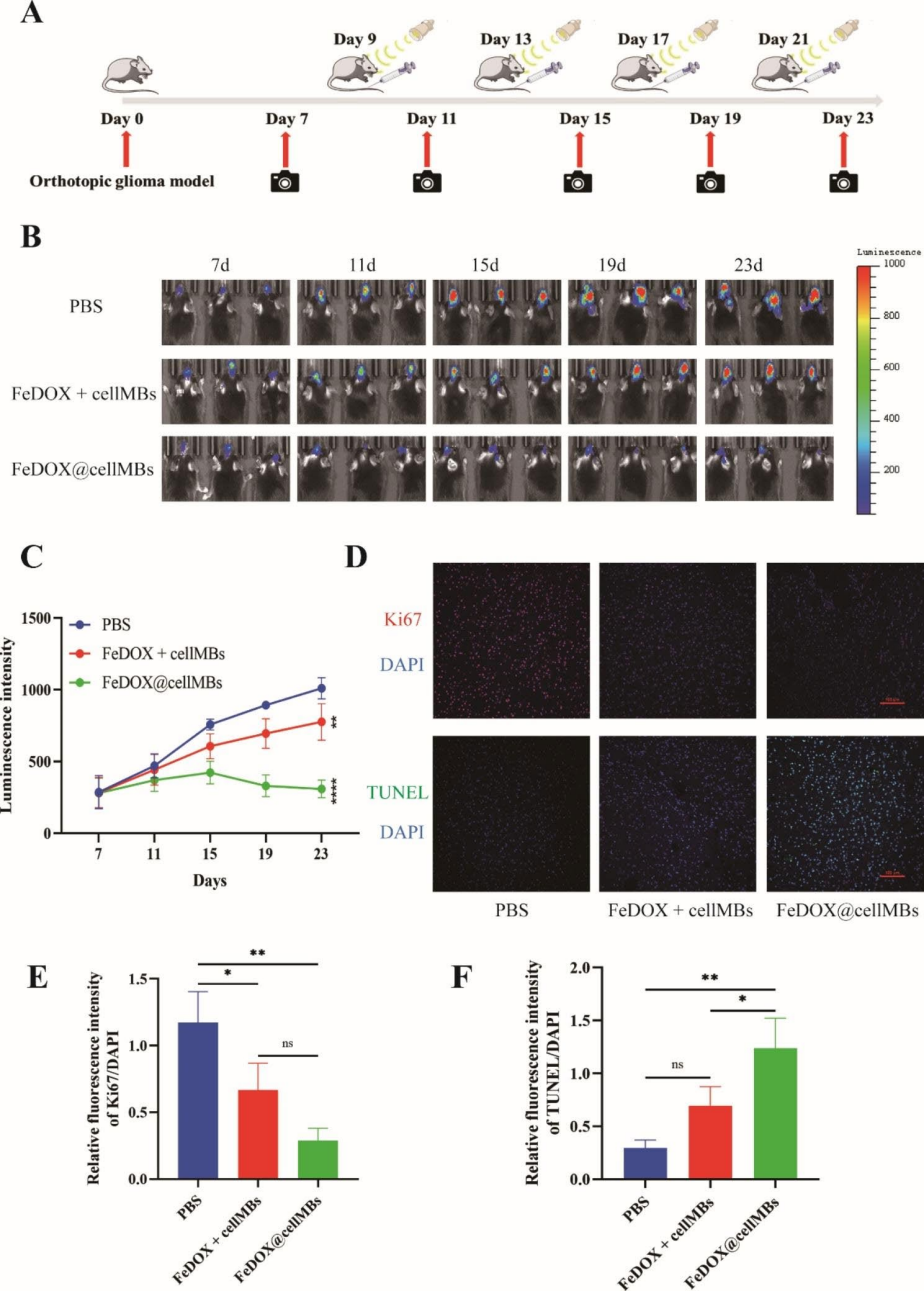
The therapeutic effect of FeDOX@cellMBs on orthotopic GBM mice. (**A**) Schematic diagram of treatment schedule. (**B**) GBM growth monitored by bioluminescence imaging (n = 3). (**C**) Semiquantitative statistic results of luminescence intensity. (**D**) Expression of Ki67 and TUNEL staining of orthotopic GBM tissue. (**E**) Semiquantitative statistic results of Ki67 expression. (**F**) Semi-quantitative statistic results of TUNEL staining. (n = 3, x ± SD. * P < 0.05, ** P < 0.01, ns: no significant)

Next, we verified the therapeutic effects of FeDOX@cellMBs on GBM cells. The FeDOX@cellMBs, the mixture FeDOX and cellMB, or PBS were injected into caudal vein, and the mice were treated with FUS and a magnetic field (Fig. 4A). Compared to PBS and the mixture of FeDOX and cellMB, the fluorescence intensity of GBM tissue in vivo was significantly lower in the FeDOX@cellMB treatment group (Fig. 4B, C). Compared with the PBS group, Ki67 expression was not decreased in the FeDOX and CellMB mixture groups, indicating that the simple mixing of the two groups cannot inhibit the growth of GBM in situ. However, Ki67 expression was significantly decreased in the FeDOX@cellMBs group. The inhibitory effect of FeDOX@cellMBs on Ki67 expression was stronger than that of the two combinations alone (Fig. 4D, E). These results indicated that FeDOX@cellMBs inhibited the proliferation of GBM cells in vivo, and the inhibitory effect was higher than that of the injection of the mixture of FeDOX and cellMBs. In addition, in situ TUNEL staining of GBM tissues showed no significant difference in the green fluorescence intensity of GBM tissues between the FeDOX and cellMBs treatment group and the PBS treatment group. At the same dose, the green fluorescence intensity of the GBM tissue in situ was significantly higher in the FeDOX@cellMBs treatment group than that in the FeDOX and cellMBs mixture treatment group (Fig. 4D, F). The results showed that FeDOX@cellMBs induced greater apoptosis in GBM cells in situ. In summary, under the combined action of FUS and magnetic field, FeDOX@cellMBs had a good therapeutic effect on GBM.

### In vivo safety of FeDOX@cellMBs

**Fig. 5:**
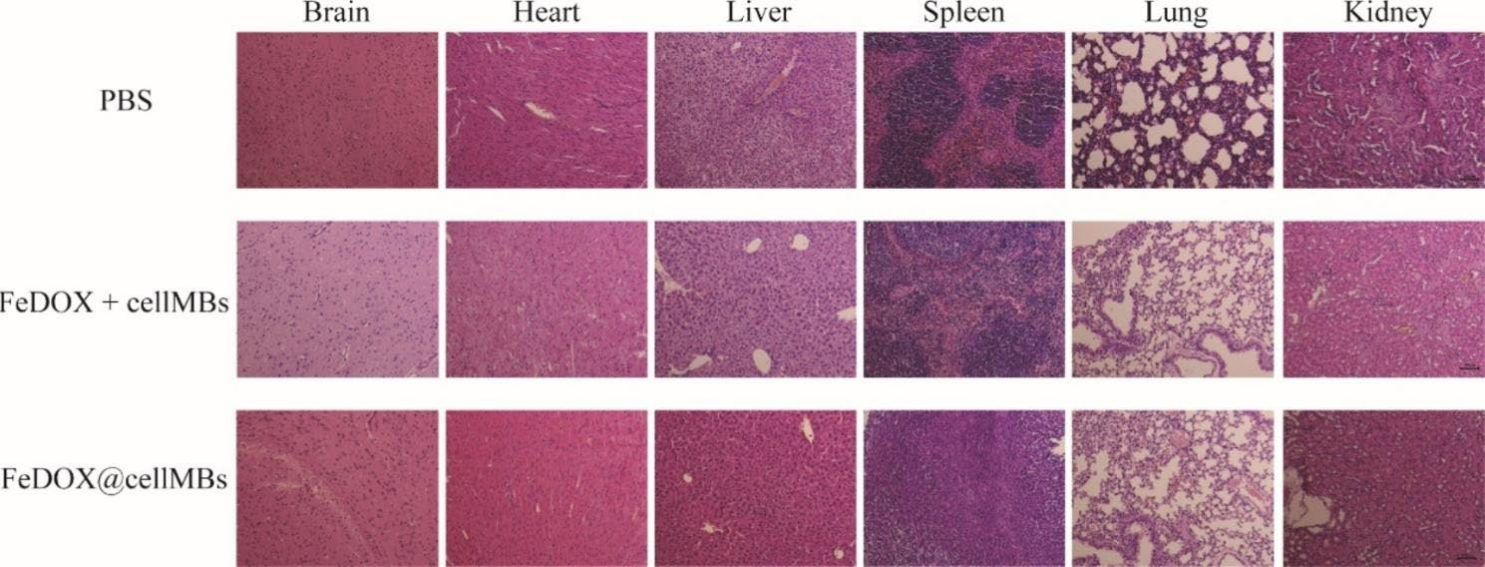
Safety of FeDOX@cellMBs in orthotopic GBM mice. H&E staining of main organs of orthotopic GBM mice (n = 3)

Finally, we examined the biosafety of the FeDOX@cellMBs. H&E staining showed that FeDOX@cellMBs did not significantly damage the brain, heart, liver, spleen, lungs, kidneys, or other major organs of GBM rats under the combined action of FUS and a magnetic field (Fig. 5). The results showed that FeDOX@cellMBs exhibited good biological safety.

## Discussion

In this study, FeDOX complexes were synthesized through natural reaction between SPIO particles and the amino and carbonyl groups of DOX. The morphology of the composite was observed using TEM, revealing a diameter of less than 100 nm. The DOX loading rate was determined to be 11.3 ± 3.7% using a UV-vis spectrophotometer. Due to its stable morphological structure, small particle size, and sufficient loading capacity, the FeDOX complex holds promise as a potential therapeutic agent for gliomas. The combination of FUS and microbubbles to reversibly open the BBB provides great facilitation and flexibility for the treatment of glioma. To better leverage this potential platform, we made interesting modifications to the microbubbles. We integrated the cell membranes from cerebral microvascular cells onto the surface of lipid microbubbles. Confocal microscopy of a mixture of stained cell membranes and ordinary lipids revealed a good fusion of the two lipid components, and gel electrophoresis confirmed the retention of cell membrane protein components in the fused lipids. Subsequently, biomimetic microbubbles containing cell membrane components were loaded with the FeDOX complex to obtain FeDOX@cellMBs. TEM analysis showed the shape of the FeDOX@cellMBs. The zeta potential test results confirmed an obvious change in the membrane potential after loading. Combined with the particle size test results, the successful preparation of FeDOX@cellMBs was indicated. Through a series of in vitro experiments, we demonstrated that FeDOX@cellMBs retained the properties of FeDOX or microbubbles without affecting their functionality. Under the influence of a magnetic field, the magnetization effect of FeDOX@cellMBs was fully manifested within 2 min. Both drug loading and cell membrane modification had no adverse effect on the response of the microbubbles to ultrasound. In addition, cell membrane modification of the microbubbles improved their in vivo stability and facilitated the effective delivery of the FeDOX complex. Treatment of a cellular model of BBB in vitro with the combination of FeDOX@cell-MBs and FUS resulted in its opening. Additionally, FeDOX was effectively taken up by glioma cells under a magnetic field after BBB permeation and showed cytotoxic effects. Following injection of FeDOX@cellMBs via the caudal vein of mice applied a combination of FUS and a magnetic field in an orthotopic mouse glioma model to examine the biological distribution and therapeutic effect of FeDOX@cellMBs on tumors. At 12 and 24 h after caudal vein injection, fluorescence in sections of various organs was observed by imaging. Consistent with the results of the in vitro experiments, the stability of FeDOX@cellMBs in vivo was significantly improved compared to that of common lipid microbubbles. Furthermore, the fluorescence intensity of the isolated organs indicated stronger DOX accumulation in the brains of the FeDOX@cellMB group than in the ordinary lipid microbubble group. Taken together our results indicate that FeDOX@cellMBs, under the combined action of FUS and a magnetic field, improved the stability of DOX in vivo and its accumulation in brain tissue. Furthermore, the results of fluorescence intensity, Ki67, and TUNEL staining confirmed the therapeutic effect of FeDOX@cellMBs on gliomas. Finally, the safety of FeDOX@cellMBs was demonstrated by biopsies of various organs. In conclusion, FeDOX@cellMBs, under the combined action of FUS and a magnetic field, not only realized the opening of the BBB but also improved the therapeutic effect on glioma with better stability, targeting, and biological safety.

Gliomas are the most common type of solid tumors in the brain. The BBB, which prevents chemotherapeutic drugs from entering the brain, is the biggest obstacle to effectively treating gliomas. The local and reversible opening of the BBB using the synergistic effect of FUS and microbubbles has been widely studied. This concept was first proposed in 2006 [14]. The delivery of chemotherapeutic drugs is considered a clinically valuable treatment for brain tumors. Deng et al. [15] constructed a magnetic nanoreactor system consisting of polylactic acid-glycolacetic acid-superparamagnetic ferric oxide (SPIO) and Vitamin C (VC). Under low-intensity FUS irradiation, VC was released on demand and locally decomposed H_2_O_2_, thus creating favorable conditions for the SPIO-based Fenton-like reaction, which greatly improved the anti-tumor effect. Wang et al. [16] developed nanoprobe-loaded MBs to monitor apoptotic cells during FUS-mediated BBB opening, providing a way to monitor and inhibit apoptotic events. However, the exact area of drug delivery to the tumor tissue and the amount of deposition could not be assessed. Direct exposure of biomimetic microbubbles to the bloodstream can be biotoxic. In this study, we extracted brain microvascular cell membranes and combined them with lipids to prepare biomimetic microbubbles containing cell membrane components. Interestingly, differential staining of the two lipid components followed by confocal microscopy revealed their good mixing and fudion. In addition, the surface of the microbubbles contained protein components of the cell membrane, which provided a solid foundation for improving their stability in vivo.

Microbubble-based drug delivery has been widely used for the treatment of various tumors [10, 17]. The bionic system FeDOX@cellMBs prepared in this study did not lose the function of each component. Functions, such as magnetic and ultrasonic responses, were demonstrated in a series of in vitro experiments. Additionally, FeDOX@cellMBs was found to effectively open an established in vitro cellular model of BBB using FUS. After opening the BBB, DOX released by FeDOX@cellMBs was taken up by glioma cells GL261 and produced an effective killing effect. The combination of FUS with a magnetic field for targeted delivery of FeDOX@cellMBs showed great potential as a treatment platform for gliomas in vitro.

Ordinary lipid microbubbles are foreign substances to the body. They are not stable in the body for long periods, which greatly reduces their clinical value. Owing to the presence of cerebrovascular endothelial cell membrane components on their surface, FeDOX@cellMBs help better evade detection by the body’s immune system. FeDOX@cellMBs were shown to be stable in mice for 12 h and could even be observed in vivo after 24 h. After dissecting the organs of the mice and observing their fluorescence intensity, DOX accumulation in the brains of mice was found to be significantly higher than that of the ordinary lipid microbubble group after 12 and 24 h under the action of a magnetic field. This is probably because FeDOX@cellMBs are more stable in vivo than ordinary lipid microbubbles. The improvement in stability also significantly enhanced the treatment effect of FeDOX@cellMBs in gliomas. Therapeutic effects were verified from the perspective of proliferation and apoptosis. The fluorescence intensity of Ki67 in the FeDOX@cellMBs group was significantly lower than that in the control group; however, the TUNEL assay showed the opposite result. Finally, H&E staining of tissue sections of the brain, heart, liver, spleen, lungs, and kidneys showed no significant damage in any organ. These results indicated that FeDOX@cellMBs had high biological safety as an effective treatment platform for gliomas.

## Conclusion

In this study, we prepared FeDOX@cellMBs by encapsulating SPIO and DOX complexes within cell membrane-modified microbubbles. The functional integrity of each component in the FeDOX@cellMBs, modified using the cerebrovascular endothelial cell membrane, was preserved. In addition, compared to regular lipid microbubbles, the FeDOX@cellMBs demonstrated significantly improved stability in vivo. Under the combined action of FUS and a magnetic field, FeDOX@cellMBs opened the BBB and released DOX accurately and completely into the brain tumor tissue, thereby maximizing the therapeutic efficacy for glioma treatment. The results indicate that FeDOX@cellMBs hold substantial potential for clinical translation when used in combination with FUS and a magnetic field, making them a promising and effective treatment platform for glioma.

### Electronic supplementary material

Below is the link to the electronic supplementary material.

Supplementary Material 1

## Data Availability

The datasets used and/or analyzed during current study are available from corresponding author on reasonable request.
