# Supplementary material for: Focused ultrasound-mediated blood-brain barrier opening combined with magnetic targeting cytomembrane based biomimetic microbubbles for glioblastoma therapy
Source: J Nanobiotechnology. 2023 Aug 26;21:297. doi: 10.1186/s12951-023-02074-z (PMC10463668; doi:10.1186/s12951-023-02074-z)

**Figure S1.** Self-developed high-intensity focused ultrasound instrument.

**Figure S1**

**A**


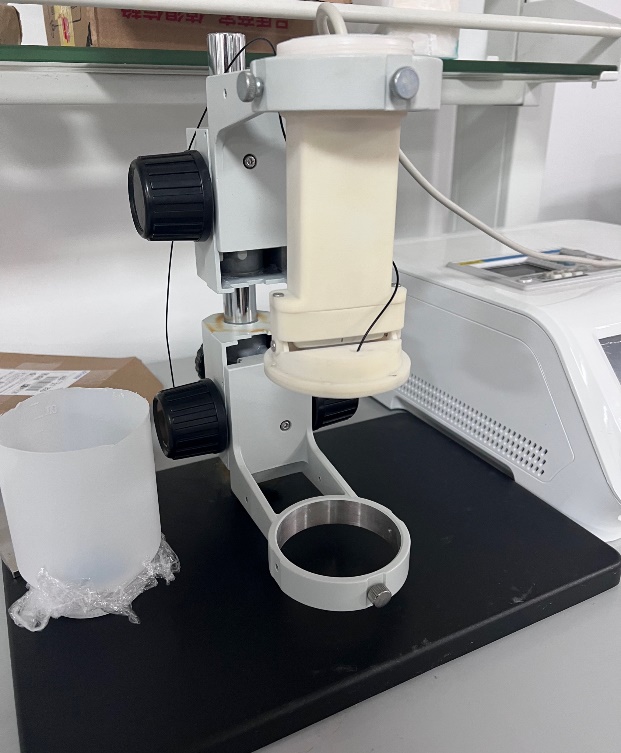


**B**


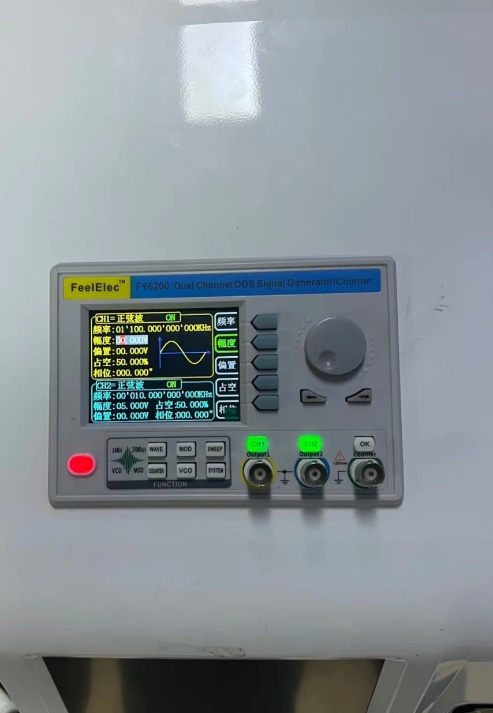


**Figure S2.** Characteristics of FeDOX@cellMBs. (A) Optical microscope images of FeDOX@cellMBs. (B) TEM images of MBs. (C) TEM images of cellMBs.

**Figure S2**


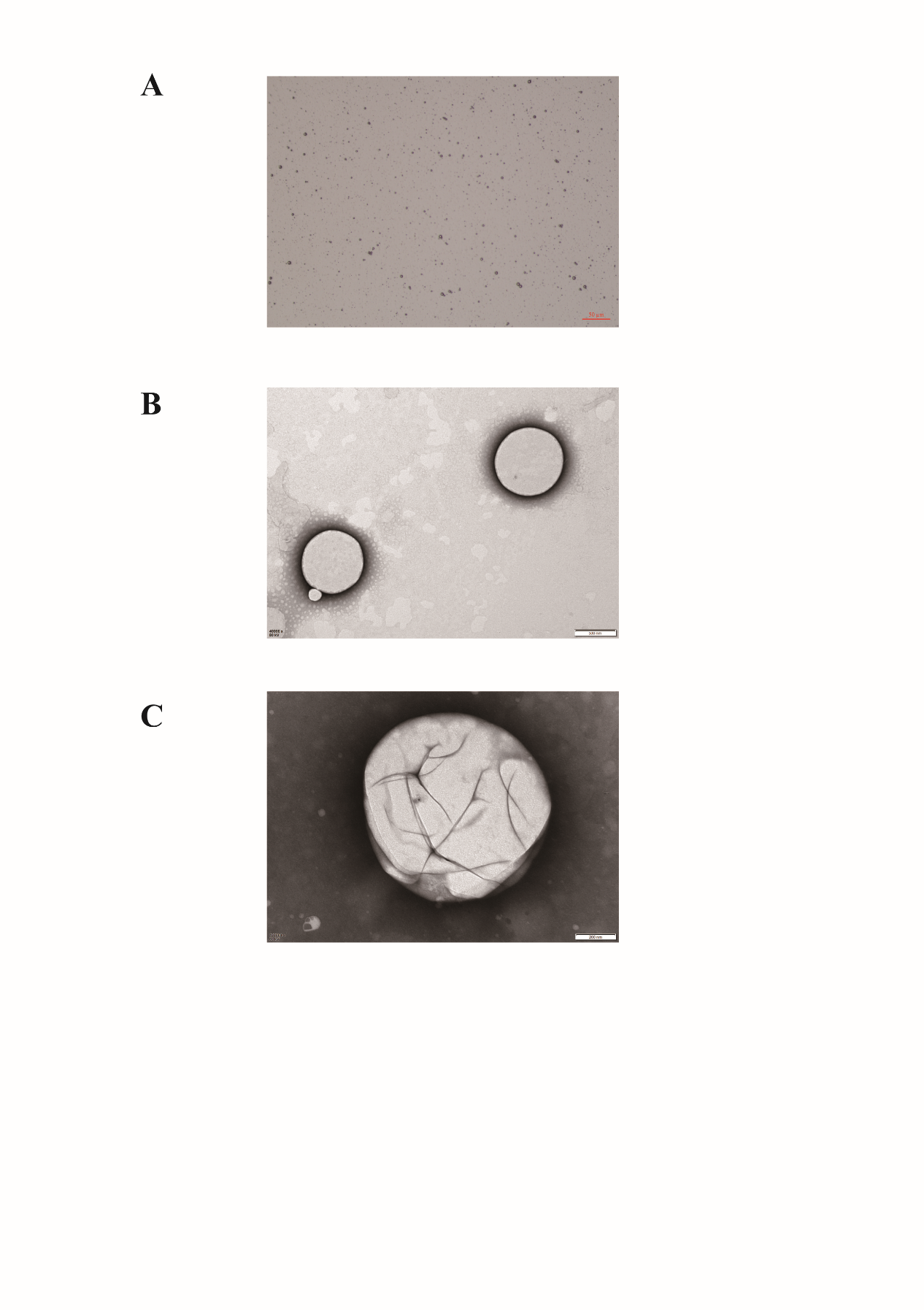

Supplement: Supplementary file 1 — Supplementary Material 1 [file 12951_2023_2074_MOESM1_ESM.docx]
